# Predictors of Hypocalcemia Post Parathyroidectomy for Primary Hyperparathyroidism; a Single Center Study

**DOI:** 10.1002/edm2.70070

**Published:** 2025-06-14

**Authors:** Khaled A. Obeidat, Nesreen A. Saadeh, Renad Msameh, Ajwad Obeidat, Omar Mar'ey, Ahmad Bakkar, Qutaiba Manasrah

**Affiliations:** ^1^ Department of Surgery, Faculty of Medicine Jordan University of Science and Technology Irbid Jordan; ^2^ Department of Medicine, Faculty of Medicine Faculty of Medicine, Jordan University of Science and Technology Irbid Jordan; ^3^ Department of Surgery King Abdullah University Hospital Irbid Jordan; ^4^ Faculty of Medicine Yarmouk University Irbid Jordan

**Keywords:** alkaline phosphatase, corrected calcium, hypocalcemia, parathyroid hormone, parathyroidectomy, primary hyperparathyroidism

## Abstract

**Background:**

Hypocalcemia is a common event after parathyroidectomy for primary hyperparathyroidism (PHPT). This study aimed to explore the incidence of hypocalcemia, determine risk factors, and identify serum biomarkers associated with the development of this condition.

**Methods:**

A retrospective study that included 116 patients with PHPT who underwent parathyroidectomy at a tertiary care facility in Jordan over 16 years (2006–2022) in this study. Patients were classified as having postoperative hypocalcemia if they developed serum calcium levels < 2.15 mmol/L within the first week following parathyroidectomy. Logistic regression analysis was performed to determine predictors of hypocalcemia. Spearman's rank correlation coefficient and ROC curves were used to assess relationships between variables as well as determine cutoffs for these predictors.

**Results:**

Of the 116 patients studied, 57.7% developed hypocalcemia after parathyroidectomy. High preoperative alkaline phosphatase (ALP), low preoperative corrected calcium, high preoperative parathyroid (PTH), and younger age were shown to be significantly higher in patients who developed hypocalcemia after parathyroidectomy. Multivariate logistic regression showed a low preoperative corrected calcium level was an independent predictor of postoperative hypocalcemia (*p* = 0.036). A high level of preoperative alkaline phosphatase was also considered an independent predictor of hypocalcemia development (OR = 1.007, 95% CI: 1.002–1.012). Patients who had pre‐operative ALP less than 208.5 U/L were unlikely to develop postoperative hypocalcemia.

**Conclusion:**

Our study identified higher preoperative ALP, lower pre‐operative corrected calcium, higher pre‐operative PTH levels, and younger age as risk factors for postoperative hypocalcemia. Preoperative ALP and preoperative corrected calcium were shown to be independent predictors of hypocalcemia development.

## Graphical abstract

This is a retrospective study that included 116 patients who underwent parathyroidectomy for primary hyperparathyroidism. The incidence of post parathyroidectomy hypocalcemia was 57.7%. Risk factors for the development of hypocalcemia were: high preoperative levels of alkaline phosphatase and parathyroid hormone, low level of preoperative corrected calcium, and younger age.

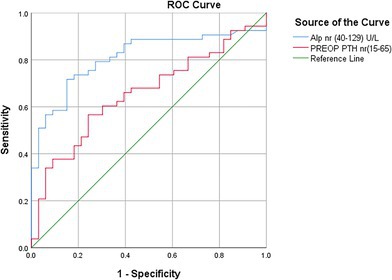

## 1 Introduction

Parathyroidectomy is a surgery to remove one or more parathyroid glands in a patient with hyperparathyroidism [1]. It is considered the only definitive treatment for primary hyperparathyroidism [2, 3]. According to the American Association of Endocrine Surgeons' guidelines (AAES), the first indication for parathyroidectomy is symptomatic primary hyperparathyroidism. However, asymptomatic patients' management is still not clear [4]. On the other hand, one of the major postoperative complications is hypocalcemia [5]. The incidence of post‐parathyroidectomy hypocalcemia in primary hyperparathyroidism is variable, with some investigators reporting a range of 12%–70% [6, 7, 8].

There was controversy over finding an agreed definition of hypocalcemia. Siyam et al. and Cooper et al. defined hypocalcemia as total serum calcium less than 2.10 mmol/L [9, 10]. At the same time, Pathy et al. assigned a hypocalcemia cutoff of 2.15 mmol/L [11]. On the other hand, Pepe et al. defined it as corrected total serum calcium levels less than 2.12 mmol/L (8.5 mg/dL); however, measurement of ionised calcium (normal values of 1.17–1.33 mmol/L) is the gold standard for diagnosis in patients with hypoalbuminemia [12, 13]. Other studies defined hypocalcemia by determining cut‐off points accompanied by specific conditions (symptomatic patients, abnormal PTH, requiring treatment, duration) [14, 15, 16].

Persistent hypocalcemia and hypophosphatemia that develop as a consequence of comprehensive re‐mineralisation of the skeletal system after PTH suppression are considered a critical complications of parathyroidectomy [17, 18, 19]. Hypocalcemia after parathyroidectomy was attributed to the rapid skeletal uptake of calcium as a result of a sudden decline in the highly elevated PTH levels. This decline was believed to induce suppression of bone resorption on top of enhanced bone formation [20]. Symptomatic hypocalcemia patients may present with seizures, tetany, numbness, tingling, perioral paresthesia, arrhythmias, cardiomyopathy, and laryngospasm [20, 21, 22].

Primary hyperparathyroidism (PHPT) is defined as long‐term excessive secretion of parathyroid hormone (PTH) as a result of an adenoma, hyperplasia, or, rarely, carcinoma. It is considered the third most common endocrine disease, with an incidence ranging from 4 to 112 per 100,000 in the USA [2, 6, 23, 24]. In addition, impaired renal function in chronic kidney disease (CKD) patients or vitamin D deficiency can cause secondary hyperparathyroidism (SHPT) [25, 26].

There are not plenty of studies discussing hypocalcemia post‐parathyroidectomy; therefore, further studies are necessary to investigate the risk factors and predictors, which are needed to improve management guidelines and prevent this complication, thereby reducing the duration of hospital stay after this operation. The importance of this study lies in enhancing the understanding of post‐parathyroidectomy hypocalcemia by examining its incidence and identifying key risk factors associated with its development, thereby contributing to the medical knowledge about this condition, particularly in the Arab world.

## 2 Methods

Our retrospective cohort study included patients with primary hyperparathyroidism who underwent parathyroidectomy surgery at the King Abdullah University Hospital (KAUH), a tertiary care facility in northern Jordan, over 16 years (2006–2022). This study was approved by the Institutional Review Board and Ethics Committee at King Abdullah University Hospital (KAUH). Data was collected using the electronic medical charts of the patients.

The primary outcome was postoperative hypocalcemia, defined as any corrected serum calcium < 2.15 mmol/L during the first postoperative week. Predictor variables included preoperative levels of calcium, PTH, phosphorus, ALP, and albumin, as well as patient demographics (age, sex), comorbidities (e.g., hypertension, diabetes, renal impairment), and pathological subtype (adenoma or hyperplasia). After surgery, patients were classified into two groups: hypocalcemic group and non‐hypocalcemic group as defined by the level of calcium mentioned above. Hyperplasia was considered a parathyroid adenoma in more than one gland.

All laboratory data were extracted from the hospital's electronic records. All patients were subjected to preoperative assessment, including serum calcium level, serum PTH level, serum phosphorus level, serum alkaline phosphatase (ALP) level, serum albumin level, and imaging findings. Postoperatively, patients were followed up for at least 4 weeks to assess changes in laboratory parameters and the occurrence of hypocalcemia. PTH, calcium, phosphorus, ALP, and albumin were measured using automated analysers according to hospital standards. Imaging findings (SESTAMIBI, ultrasound, CT) were reviewed from radiology and nuclear medicine reports. Serum calcium levels were adjusted for serum albumin using Payne's formula: (Serum calcium (mmol) + 0.02 × 40‐albumin (g/L)). This study included all patients with primary hyperparathyroidism who underwent parathyroidectomy at KAUH during the period (2006–2022). We excluded patients who still had persistent hyperparathyroidism after surgery and patients who missed follow‐up for at least 4 weeks post‐surgery.

## 3 Statistical Analysis

Descriptive statistics were reported as percentages for categorical variables and medians with interquartile ranges (IQR) for continuous variables. The Kolmogorov–Smirnov test confirmed non‐normal distribution of quantitative variables. The Mann–Whitney *U* test was used for continuous variables, and Fisher's exact test was used for categorical variables. Multivariable logistic regression was performed to identify independent predictors of hypocalcemia, reporting odds ratios (ORs) and 95% confidence intervals (CIs). Spearman's rank correlation assessed associations between continuous variables. Receiver operating characteristic (ROC) analysis evaluated predictive accuracy, with cut‐offs determined by Youden's index. Missing data were minimal (< 5%) and addressed by complete case analysis. All analyses were conducted using IBM SPSS version 26, with significance set at *p* < 0.05. All variables were treated as continuous unless otherwise specified.

Selection bias was minimised by including all eligible patients during the study period without sampling. Information bias was addressed by relying on objective laboratory values and imaging findings from standardised institutional protocols.

## 4 Results

During the study period, a total of 116 patients who underwent a parathyroidectomy as a result of parathyroid adenoma or hyperplasia were studied (Table 1). Eighty‐one were females (69.8%), and the median age was 46 years (interquartile range [IQR] 21 years). Sixty‐seven (57.7%) patients developed post‐parathyroidectomy hypocalcemia. Most of the patients had adenoma (86.1%) in comparison with hyperplasia (13.9%). Regarding comorbid characteristics, a good proportion of patients had comorbidities: hypertension (31%), diabetes mellitus (22.4%), renal stones (12.1%), and renal impairment (18.1%).

**TABLE 1:** Baseline characteristics by hypocalcemia status.

	Non‐hypocalcemic (*N* = 49)^a^	Hypocalcemic (*N* = 67)^a^	*p*
	Count (%) or median (IQR)	Count (%) or median (IQR)	
Pathology			
Adenoma	46 (93.9%)	53 (80.3%)	0.038
Hyperpalsia	3 (6.1%)	13 (19.7%)	
Age (year)	52 (42–60)	42 (34–52)	0.001
Gender			
Female	30 (61.2%)	51 (76.1%)	0.084
Male	19 (38.8%)	16 (23.9%)	
Hypertension, yes (%)			
No	28 (57.1%)	52 (77.6%)	0.019
Yes	21 (42.9%)	15 (22.4%)	
Diabetes mellitus, yes (%)			
No	37 (75.5%)	53 (79.1%)	0.647
Yes	12 (24.5%)	14 (20.9%)	
Renal stones, yes (%)			
No	41 (83.7%)	61 (91.0%)	0.229
Yes	8 (16.3%)	6 (9.0%)	
Renal impairment, yes (%)			
No	43 (87.8%)	52 (77.6%)	0.161
Yes	6 (12.2%)	15 (22.4%)	
Weight by category			0.613
≤ 2 g	24 (53.3%)	30 (48.4%)	
> 2 g	21 (46.7%)	32 (51.6%)	
Weight of parathyroid (g)	2.0 (1.0–3.7)	2.16 (0.74–3.75)	0.772
Pre‐op PTH (pg/mL)	218.0 (148.9–300.2)	390 (225–1145)	< 0.001
Pre‐op corrected calcium (mmol/L)	2.7 (2.6–2.9)	2.58 (2.40–2.73)	< 0.001
Pre‐op alkaline phosphatase (IU/L)	104 (87–187)	324 (172–629)	< 0.001
Pre‐op phosphorus (mmol/L)	0.83 (0.72–0.92)	0.82 (0.61–1.37)	0.762
Post‐op PTH (pg/mL)	28.5 (11.0–58.2)	32.2 (12.3–85.3)	0.746
Post‐op phosphorus (mmol/L)	1.00 (0.86–1.09)	1.1 (0.8–1.3)	0.421
Creatinine (μmol/L)	69 (55–84)	59 (48–115)	0.553
Creatinine clearance (mL/min)	101.4 (77.1–140.8)	90 (66–153)	0.414

^a^ *N* value varies slightly for surgery type variable, *n* = 109, pathology, *n* = 115 and weight of parathyroid, *n* = 107.

On univariate analysis (Table 1), the median age of patients who developed hypocalcemia was significantly lower by 10 years (*p* = 0.001). Age was also a significant risk factor when performing Spearman's rank correlation coefficient but didn't reach significance on multivariate analysis (Table 2). Among patients who had hyperplasia, the risk of hypocalcemia development was approximately 3 times higher than that of non‐hypocalcemic patients (*p* = 0.038). Patients who had hypertension were approximately 2 times less likely to develop hypocalcemia (*p* = 0.019). However, they proved to be insignificant on multivariate analysis.

**TABLE 2:** Multivariate logistic regression identifying independent predictors of postoperative hypocalcemia.

Multivariate logistic regression (hypocalcemic vs. non‐hypocalcemic)				
	Sig.	OR	95% CI for OR	
			Lower	Upper
Age	0.845	1.005	0.960	1.051
HTN	0.381	1.767	0.494	6.319
PREOP CA (corrected)	**0.036**	0.063	0.005	0.834
PREOP PTH	0.597	1.000	0.999	1.001
Alp	**0.011**	1.007	1.002	1.012
Constant	0.125	262.758		

*Note:* Bold values indicate statistical significance.

**FIGURE 1:**
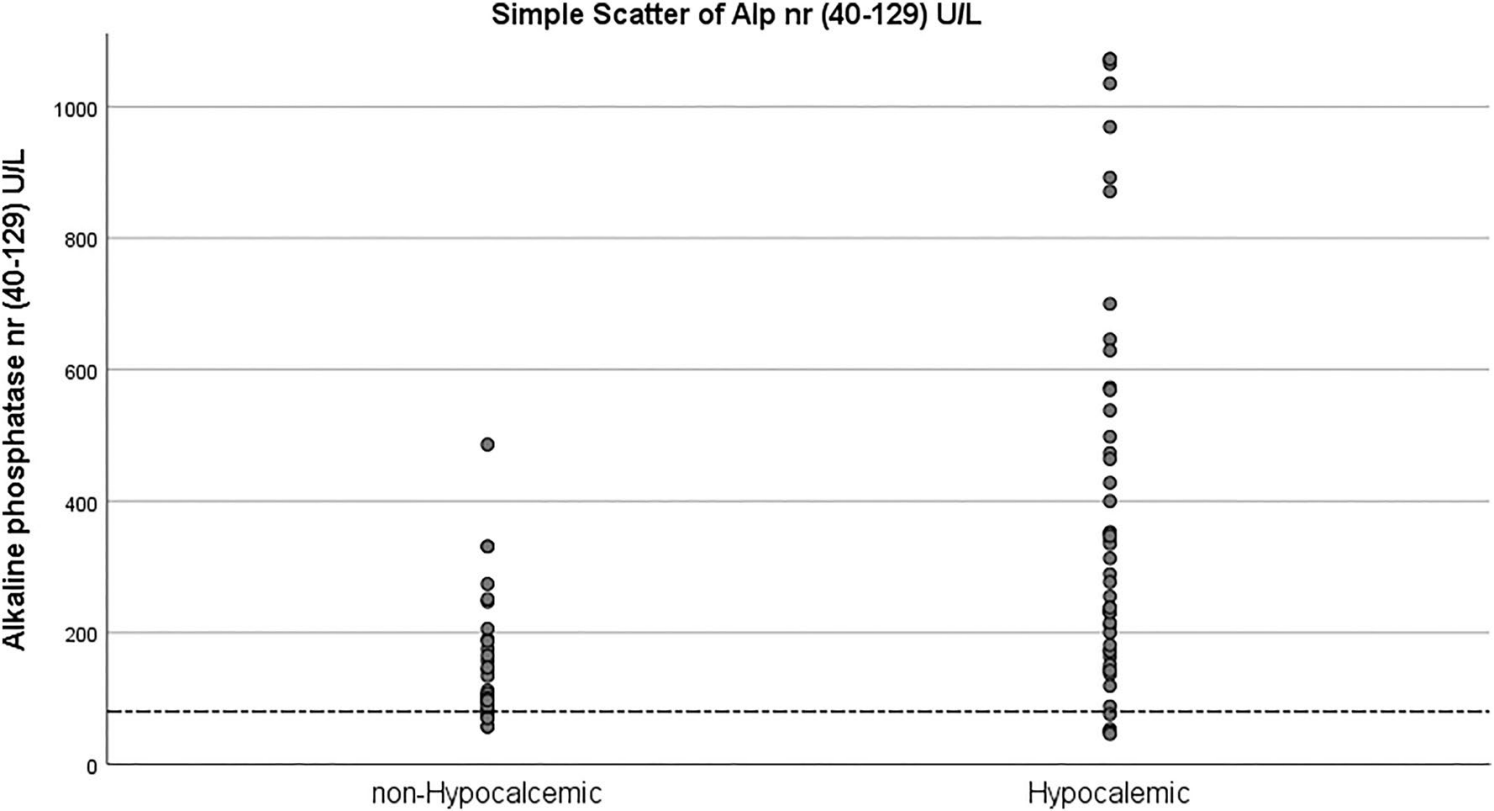
Scatter plot of preoperative alkaline phosphatase (ALP) levels in hypocalcemic versus non‐hypocalcemic patients.

**FIGURE 2:**
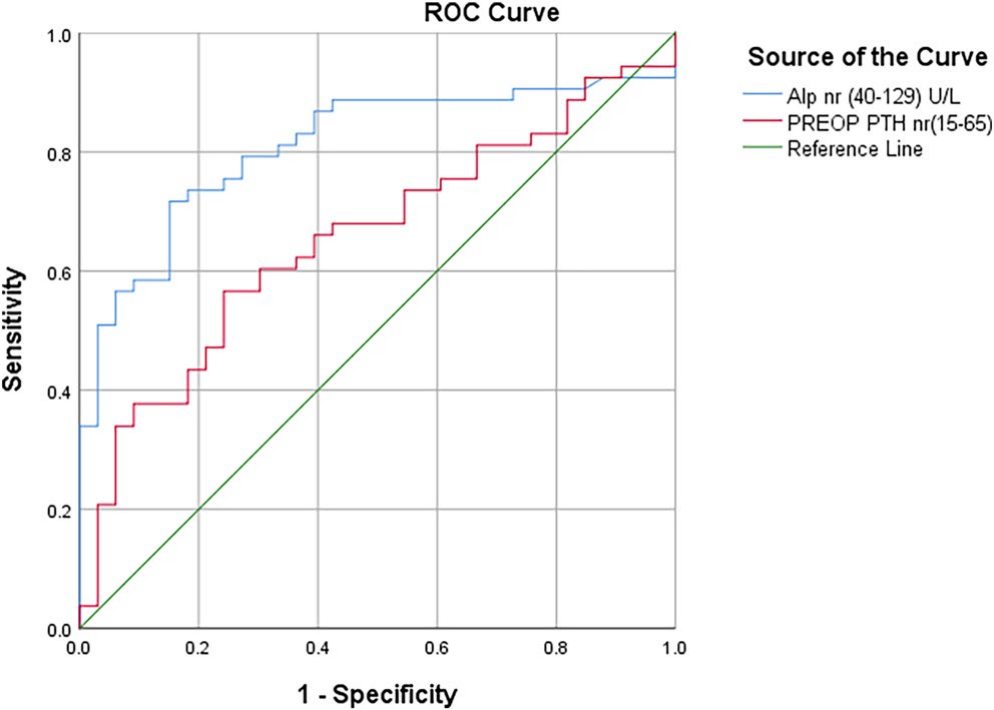
ROC curve comparing the predictive value of preoperative ALP and PTH for postoperative hypocalcemia.

Hypocalcemic patients were detected to have statistically significant higher levels of pre‐op alkaline phosphatase (*p* < 0.001). This association can be clarified as a significant negative correlation between alkaline phosphatase and postoperative calcium level (*r* = −0.627, *p* < 0.001). Notably, higher alkaline phosphatase was an independent predictor for hypocalcemia development (OR = 1.007, 95% CI: 1.002–1.012) using different statistical analyses (Figure 1 and Tables 1, 2, and 4). Figure 1 shows the scatter plot for alkaline phosphatase and demonstrates that hypocalcemic patients had higher levels of preoperative alkaline phosphatase. In contrast, Figure 2 contains ROC analysis, which shows that preoperative alkaline phosphatase has the highest sensitivity (71.7%) and specificity (84.8%) at 208.5 U/L cut‐off level. The area under the curve (AUC) was 0.810 (95% CI: 0.717–0.903) (Table 3).

**TABLE 3:** Diagnostic performance of ALP and preoperative PTH for predicting postoperative hypocalcemia.

Test result variable(s)	Area	Std. error	Asymptotic Sig.	Asymptotic 95% Confidence Interval		Cut‐off value	Sensitivity	Specificity
				Lower Bound	Upper Bound			
Alp nr (40–129) U/L	0.810	0.047	0.000	0.717	0.903	208.5	71.7%	84.8%
PREOP PTH nr(15–65)	0.655	0.059	0.016	0.539	0.770	353.9	56.6%	75.8%

In addition, lower preoperative corrected calcium levels were shown to be independent predictors of postoperative hypocalcemia. This was observed using different analyses where hypocalcemic patients were shown to have a lower median of preoperative corrected calcium levels compared with patients who did not (2.58 vs. 2.7 mmol/L, *p* < 0.001) (Tables 1 and 2). In addition to showing a positive correlation (*r* = 0.351, *p* < 0.001) between preoperative corrected calcium and postoperative calcium levels (Table 4).

**TABLE 4:** Spearman's correlation between clinical/laboratory parameters and postoperative calcium levels.

	Weight of parathyroid (in grams)	Age	PREOPALB nr (35–52)	CORRECTED CA nr (2.15–2.5)	PREOP PTH nr (15–65)	Alp nr (40–129) U/L
*r*	−0.018	0.351	0.153	0.463	−0.476	−0.627
*p*	0.850	**< 0.001**	0.135	**< 0.001**	**< 0.001**	**< 0.001**

*Note:* Bold values indicate statistical significance.

Regarding pre‐operative PTH, levels were significantly higher in hypocalcemic patients (*p* = < 0.001), which was illustrated by a negative correlation (*r* = −0.476, *p* < 0.001) between pre‐operative PTH and post‐operative calcium level (Tables 1 and 4). However, in multivariate logistic regression, pre‐operative PTH was not identified as an independent predictor of postoperative hypocalcemia (95% CI: 0.999–1.001) (Table 2).

Figure 2 presents the ROC curve of preoperative PTH. The optimal diagnostic value was 353.9 pg/mL, with sensitivity and specificity of 56.6% and 75.8%, respectively, and an area under the curve (AUC) of 0.655 (95% CI: 0.539–0.770) (Table 3).

In Table 5, a multivariate linear regression model was constructed to identify the predictors of postoperative calcium levels. Corrected calcium level (*β* = 0.221, *p* = 0.014), preoperative PTH level (*β* = −0.353, *p* = 0.001), and ALP level (*β* = −0.369, *p* = 0.001) were all found to be significant independent predictors (Table 5). An equation can be formulated to predict postop calcium as follows: Postop calcium (mmol/L) = 0.221 × preoperative CC (mmol/L) – 0.369 × preoperative ALP (U/L)—0.353 × preoperative PTH (pg/mL) + 1.695 (*R* = 0.705, *R* ^2^ = 0.497, *p* < 0.001). The model accounted for approximately 50% of the variance in calcium levels (*R* ^2^ = 0.497, *p* < 0.001), with ALP showing the strongest negative association.

**TABLE 5:** Multivariate linear regression analysis for predictors of postoperative calcium level.

	Standardised coefficients	*p*	95.0% confidence interval for B	
	Beta		Lower bound	Upper bound
(Constant)	1.695	0.000	1.255	2.135
CORRECTED CA nr(2.15–2.5)	0.221	0.**014**	0.042	0.362
PREOP PTH nr(15–65)	−0.353	0.**001**	0.000	0.000
Alp nr (40–129) U/L	−0.369	0.**001**	0.000	0.000

*Note: R* = 0.705, *R* ^2^ = 0.497, *p* < 0.001. Bold values indicate statistical significance.

No significant differences were noted between the two groups in postoperative phosphorus, PTH, serum creatinine, or creatinine clearance. Additionally, parathyroid gland weight and weight category (≤ 2 g vs. > 2 g) were not significantly associated with hypocalcemia (*p* = 0.772 and *p* = 0.613, respectively). There were no significant differences between the two groups in terms of sex (*p* = 0.084), diabetes mellitus (*p* = 0.647), renal stones (*p* = 0.229), or renal impairment (*p* = 0.161).

## 5 Discussion

Postoperative hypocalcemia is one of the most common and most important complications following parathyroidectomy for primary hyperparathyroidism, with an incidence ranging from 10% to 70% in previous reports [7, 8, 24, 27, 28, 29, 30, 31, 32, 33, 34, 35, 36, 37, 38]. In our study, the incidence of hypocalcemia was 57.7%, but most of these were transient. The wide variation can be explained by different definitions of hypocalcemia as well as variations in patients' characteristics among different studies. Several reports have looked for risk factors that can contribute to the development of hypocalcemia after parathyroidectomy. The most common risk factors that were studied in these reports were levels of preoperative ALP, PTH, and calcium, weight and number of resected parathyroid, percent of the drop in intraoperative PTH level, radiological changes in bone, and age of the patient [20, 21, 22, 24, 26, 30, 31, 32, 33, 34, 35, 36, 37, 38, 39, 40, 41, 42, 43, 44, 45, 46, 47, 48, 49].

Our data showed that preoperative parathyroid hormone and alkaline phosphatase were significantly higher in patients who developed hypocalcemia. Whereas preoperative corrected calcium and age were shown to be significantly lower. Regarding preoperative calcium level, Steen et al. and Mu et al. also found that having normal or only minimally elevated preoperative calcium was an independent risk factor for postoperative hypocalcemia after parathyroidectomy for primary hyperparathyroidism [49, 50]. Similar findings were found in other studies in patients with secondary hyperparathyroidism [44, 45, 47].

In the present study, we found elevated preoperative ALP levels to be a significant predictor of postoperative hypocalcemia, and that was proved by more than one statistical analysis method. In addition, ROC curve analysis showed that ALP has the highest sensitivity and specificity for predicting postoperative hypocalcemia at a cutoff of 208 U/L with an area under the curve of 0.81. Loke et al. reported that preoperative serum ALP was shown to have a negative correlation with postparathyroidectomy calcium levels; after general linear model (GML) analysis, ALP was the only independent predictor for postoperative hypocalcemia, concluding that patients who had preoperative ALP less than 340 U/L were unlikely to develop symptomatic postoperative hypocalcemia [30]. Moreover, Sun et al. also defined preoperative ALP as an independent risk factor for postoperative hypocalcemia with an optimal cut‐off point of 269 U/L [31]. Several studies investigated preoperative ALP levels and found that elevated ALP was significantly associated with post‐parathyroidectomy hypocalcemia in patients with primary or secondary hyperparathyroidism [21, 24, 33, 38, 44, 45, 47, 48, 51]. Elevated preoperative serum ALP levels can be imputed to their impact on regulating bone mineralization (bone formation) in addition to reflecting the amount of osteoclast activity and bone resorption [20, 52].

In agreement with our findings, higher preoperative PTH levels were shown to be statistically significantly associated with the development of hypocalcemia. Additionally, when ROC was performed by Jakubauskas, the 45 pmol/L (424.3 pg/mL) cut‐off value of the preoperative PTH level was reported to have the highest sensitivity and specificity to predict the development of postoperative hypocalcemia [40]. Moreover, Kald et al. established that postoperative hypocalcemia development was significantly associated with higher PTH levels at a cutoff of 35 pmol/L (330 pg/mL). These results may be considered a guide to determining the proper time for a hospital stay after a parathyroidectomy. Higher preoperative PTH (> 35 pmol/L) gives indications for a longer postoperative hospital stay (> 23 h) until the normal calcium level is reached [41].

In comparison with preoperative PTH, preoperative ALP was considered a better predictor of postoperative hypocalcemia. This can be demonstrated by the difference in their biological half‐lives (8 h vs. 4 min for PTH) [53, 54].

Based on our analysis, younger patients were found to be significantly associated with the risk of postoperative hypocalcemia development. A controversial relationship was found between the hypocalcemia development after parathyroidectomy and the age of the patient at surgery time. In agreement with our findings, Zuberi et al. stated that younger age was significantly associated with the development of hypocalcemia after parathyroid resection [32]. In addition, young age was reported as a risk factor for postoperative hypocalcemia in patients with secondary hypoparathyroidisms [22, 26, 44, 45].

In contrast, other studies revealed that older patients had a significantly higher risk for hypocalcemia development [33, 42, 46]. These results may be explained by the association between older age and vitamin D deficiency, decreased 1 alpha‐hydroxylase enzyme activity, and low dietary calcium intake, which all can lead to a decrease in the serum calcium level and bone complications [20, 33].

Regarding the number of resected glands, it was associated with postop hypocalcemia in univariate analysis but not in the other analyses, indicating its insignificance as a predictor. However, Mittendorf et al. found that patients with primary hyperparathyroidism who underwent parathyroidectomy for one or two glands had significantly higher postoperative calcium levels than patients who underwent subtotal parathyroidectomy; similar findings were reported by Torer, Chia, Kald, and Zuberi et al. [26, 32, 34, 41, 43].

Additionally, we did not find that the weight of the resected parathyroid gland was associated with hypocalcemia after parathyroidectomy. Whereas, several studies found that higher parathyroid gland weight was associated with an increased risk of postoperative hypocalcemia. This can be attributed to the negative feedback effect of heavier hyperfunctional glands on other normal glands [26, 33, 35, 38, 40].

This study contributes to regional data on PHPT. Its strengths include a 16‐year dataset, biochemically defined endpoints, and robust statistical modelling. However, its limitations include its retrospective nature, the small size of the sample, and the lack of certain data about possible confounding factors like concomitant vitamin D deficiency, preoperative bone mineral density, preoperative use of bisphosphonates and vitamin D, and perioperative administration of vitamin D and/or calcium.

## 6 Conclusion

Our data provide insight into the evidence, which suggests that hypocalcemia development after parathyroidectomy for primary hyperparathyroidism is associated with multiple preoperative biomarkers. Our study concluded that pre‐operative corrected calcium, ALP, PTH, and age are risk factors for hypocalcemia development post‐parathyroidectomy. Furthermore, we tried to determine the optimum cutoff for pre‐operative ALP as a predictor of hypocalcemia development. More extensive multicentric research is needed to clarify the debated matters about this topic.

## Author Contributions

**Khaled A. Obeidat:** conceptualization (equal), methodology (equal), project administration (equal), supervision (equal), visualization (equal), writing – original draft (equal), writing – review and editing (equal). **Nesreen A. Saadeh:** conceptualization (equal), methodology (equal). **Renad Msameh:** data curation (equal), resources (equal). **Ajwad Obeidat:** formal analysis (equal), software (equal), writing – original draft (equal). **Omar Mar'ey:** formal analysis (equal), software (equal), writing – original draft (equal). **Ahmad Bakkar:** formal analysis (equal), software (equal), writing – original draft (equal). **Qutaiba Manasrah:** formal analysis (equal), software (equal), writing – original draft (equal).

## Ethics Statement

The study was performed according to the Helsinki Declaration and institutional review board approval was obtained.

## Conflicts of Interest

The authors declare no conflicts of interest.

## Data Availability

Data will be available upon request.
